# Clinical and Radiographic Evaluation of Applying Atorvastatin 1.2% Bio Adhesive with Plasma Rich in Growth Factor (PRGF) for Treatment of Mandibular Class II Furcation Defects: a Randomized Clinical Trial 

**DOI:** 10.30476/DENTJODS.2021.84581.1090

**Published:** 2022-06

**Authors:** Niloofar Jenabian, Sakine Mohammadpour, Sina Haghanifar, Sohrab Kazemi, Mahmood Hajiahmady

**Affiliations:** 1 Oral Health Research Center, Health Research Institute, Babol University of Medical Sciences, Babol, Iran; 2 Dept. of Periodontics, North Khorasan University of Medical Sciences, Bojnurd, Iran; 3 Oral Health Research Center, Health Research Institute, Oral and Maxillofacial Radiology, Babol University of Medical Sciences, Babol, Iran; 4 Cellular and Molecular Biology Research Center, Health Research Institute, Babol University of Medical Sciences, Babol, Iran; 5 Dept. of Biostatistics and Epidemiology, Babol University of Medical Sciences, Babol, Iran

**Keywords:** Furcation defect, Growth factors, Atorvastatin

## Abstract

**Statement of the Problem:**

Molar teeth with furcation involvement are one of the most common problems in patients with periodontal disease. Regeneration methods are of the most controversial treatment strategies for these lesions.

**Purpose:**

The purpose of this study was to determine the effect of plasma rich in growth factors (PRGF) with 1.2% Atorvastatin (ATV) in the treatment of furcation involvement of mandibular molars.

**Materials and Method:**

The present randomized clinical trial was conducted on 15 patients with moderate periodontitis and class II furcation involvements; 24 defects were located in four groups of six, including debridement, ATV1.2%, PRGF, PRGF with ATV1.2%. The parameters of vertical probing depth (VPD), vertical clinical attachment level (VCAL), gingival index (GI), horizontal probing depth (HPD) and gingival recession (GR) were measured at baseline (T0), immediately before surgery (T1), 3 (T2), and 6 (T3) months after surgery. Moreover, the bone conditions were evaluated by digital subtraction radiography before and six months after surgery. Data were analyzed using SPSS23 software.

**Results:**

No significant difference in radiographic parameters was observed among the groups (*p*= 0.08). There was no significant difference in the mean levels of VPD, VCAL and HPD among the groups at different times (*p*<0.05). Comparison of clinical parameters of VPD, VCAL and GI in the treatment groups compared to the baseline showed a significant improvement in each group (*p*< 0.05) but there was no significant difference among different groups (*p*< 0.05).

**Conclusion:**

The use of PRGF with ATV 1.2% in grade II furcation involvement in mandi-bular molars was effective in the improvement of clinical and radiographic parameters six months after treatment, but this effect revealed no difference in comparison with the other groups.

## Introduction

Periodontal disease is a multifactorial condition that causes pocket formation, clinical attachment loss, and bone resorption. Furcation involvement is defined as the destruction of periodontal tissue in the interradicular area of bone in multirooted teeth that occurs due to plaque-associated periodontal diseases [ 1 - 3 ]. Some of the features of furcation defects clinically include attachment loss in both vertical direction along the root and horizontal direction to the interior of the furcation area [ 4 ]. Concerning the complex morphology of the region, the furcation involvements is considered as one of the most challenging aspects of periodontal treatment and causes problems in the success of periodontal treatments [ 5 - 6 ]. 

Although the prognosis of furcation-involved molars has not been reported to be hopeless, the presence of periodontal disease in these areas will significantly increase tooth loss due to limited access to the dentist and patient. The treatment of periodontal lesions in these areas is one of the most difficult problems facing general dentists and periodontists, and so far, no completely predictable and ideal treatment has been provided for this condition [ 7 ]. 

In most cases, the outcome of the treatment is subjected to variety of causative factors including tooth-related (anatomical aspects) and patient-related factors (such as diabetes, smoking, stress, and so on) [ 2 ]. 

Many therapeutic strategies, including the use of autogenous bone grafts and bone substitute materials [ 7 - 11 ] and growth factors [ 1 , 6 , 12 - 15 ] have been suggested to promote regeneration of periodontal tissues. 

The treatment of periodontal furcation lesions in multi-root teeth is a major challenge in regenerative medicine [ 6 - 7 , 12 , 16 ]. The goal of periodontal treatment is to regenerate the tissues lost caused by periodontal disease. Lesions of the periodontal ligament and surrounding alveolar bone may result from infections of the periodontium or tissue of the dental pulp [ 17 ]. 

Many approaches to regenerative periodontal therapy are based on the use of growth factors and bone regeneration materials [ 7 - 8 , 18 - 19 ], guided tissue regeneration [ 7 , 17 , 20 ] and enamel matrix derivatives [ 20 ], in grade II furcation involvements. Statins are one of the materials that have been tested for the regeneration therapies [ 21 - 22 ].

Biologically active endogenous proteins offer a novel advance to tissue regeneration. In 1999, Anitua [ 23 ] proposed a technique for preparing plasma rich in growth factors (PRGF). This autologous preparation is enriched with biological mediators that accelerate regeneration of both hard and soft tissues. PRGF contains a high concentration of a platelet-derived growth factor, insulin-like growth factor, and fibroblast growth factor and has the least concentration of pro-inflammatory interleukins considering the absence of leukocytes [ 16 ]. 

The PRGF is a second-generation system, similar to platelet-rich plasma (PRP), which is used to obtain plasma proteins and platelets and requires less venous blood, is safe and easier to use, takes less time to prepare, and leads for faster healing [ 24 ]. It is reported that periodontal pocket improvement is related to increasing gingival epithelial attachment on the root surface through cell differentiation and proliferation. The growth factors released from the PRGF induce some bioprocesses such as cell proliferation, migration, and differentiation [ 24 ]. 

Mansouri *et al*. [ 6 ] employed bovine porous bone mineral plus PRGF for the treatment of grade II furcation and reported a significant reduction in the relative vertical clinical attachment level, horizontal clinical attachment level, and gingival index to baseline. 

Statins are categorized as one of the lipid lowering drugs, which help reduce cholesterol levels by inhibiting 3-hydroxy-3-methylglutaryl coenzyme A (HMG-CoA) reductase. Moreover, Statins help promote angiogenesis and inhibiting metastasis by increasing the production of BMP-2 and hence, assisting in osteoblastic differentiation. They have different functions such as anti-inflammatory, immune-modulatory, antioxidant, antithrombotic and endothelium stabilization roles. Osteoblastic differentiation and anti-inflammatory are those actions of statins, which can be employed to treat periodontal disease [ 25 - 27 ]. 

Atorvastatin (ATV ) has favorable effects on alveolar bone loss and tooth mobility and improves clinical parameters like probing depth reduction and clinical attachment level (CAL) gain when used as an adjunct to scaling and root planning (SRP) in the treatment of class II furcation defects [ 21 - 22 ].

To the best of authors’ knowledge, no study has evaluated the effect of ATV gel combined with PRGF in the treatment of class II furcation lesions yet. Therefore, the present study was conducted to assess the effect of combined PRGF and ATV 1.2% on the treatment of furcation involvement in first and second molars of mandible.

## Materials and Method

The current study was approved by the Ethical Committee of University (MUBABOL.REC.1396.25). It is also registered in the WHO clinical trial registry, branch of the Islamic Republic of Iran (IRCT: 20100427003813 N7) and a written informed consent was obtained from all subjects after providing a complete description of the study interventions.

### Study design and eligibility criteria

This study was planned as a randomized, double blind (patient, clinician) clinical trial. Fifteen patients with twenty-four defects referred to the Periodontology Department of Babol University of Medical Sciences who were diagnosed with a moderate chronic periodontitis with buccal or lingual Grade II furcation involvement of the first or second vital mandibular molars and had ≥3mm vertical probing depth (VPD) were enrolled in this study. The sample size was estimated to be 24 using the Altman plot with test power of 80% and the type I error of 0.05.

Exclusion criteria were defined as (1) any systemic disease, (2) consumption of medications interfering with periodontal wound healing (such as corticosteroids, immunosuppressive, and anti-inflammatory drugs), (3) smoking habit, (4) the presence of cavity or filling in furcation area, (5) teeth with anatomical complications such as cervico-enamel projection and/or bifurcation ridges and concavity, (6) Miller’s mobility of Grade II and more in involved teeth, (7) the need for antibiotic prophylaxis prior to surgery, (8) any history of allergies to the predetermined materials and any contraindication for surgical procedure, (9) the presence of the periapical lesions in radiography and endodontic treatment, and (10) the unwillingness of the patient to do a periodontal surgery or any of uncooperative patient after initial periodontal treatment.

### Randomization and blindness

The lesions were randomly divided and coded into four groups of six named as (1) PRGF, (2) ATV 1.2% (Chemidarou, IRAN), (3) ATV 1.2% bio adhesive with PRGF, and (4) the control group. The clinical variables were measured at baseline, immediately before surgery, 3, and 6 months after surgery around each tooth by a periodontist who was unaware of the treatment using a Williams probe (HU-Friedy; Chicago; IL; USA). A clinician who performed the surgery was different from the one who measured the parameters. A dentomaxillofacial radiologist who was blind to the study reported the osseous changes. 

### Study protocol

Informed consent forms were filled and signed by all recruited patients. Oral hygiene instruction and SRP was performed for all patients for preoperative management of bacterial biofilm level. The occlusion was checked and adjusted if needed. All surgical procedures were performed by one surgeon. The surgical procedure included the administration of local anesthesia (Lidocaine 2% with Epinephrine 1:80, 000), intrasulcular incision with mucoperiosteal flap elevation, debridement of granulation tissue, sub gingival SRP, and rinsing with normal saline. The lesions were then randomly assigned to one of four treatment groups. In the control group (group 1), debridement of the lesion was performed alone. In the second group, 1 ml of activated PRGF was applied to the lesion walls and root surfaces. In the third group, the ATV bio adhesive alone was inserted into the furcation lesion. In the fourth group, first the ATV bio adhesive was placed in a container containing PRGF (PRGF had the ability to remain in the lesion due to the suitable consistency of the ATV bio adhesive) to absorb some of it in the bio adhesive, and finally placed inside the lesion. The PRGF was then placed on the lesion site filled with ATV. Next, the flap was returned coronally in all groups, and sutured with silk suture 3-0.

After surgery, the patients were instructed to use 0.12% chlorhexidine mouthwash (Emad pharmaceutical Co.; Isfahan; Iran) twice daily for four weeks. Ibuprofen (400mg, Hakim Pharmaceutical Co.; Tehran; Iran) was administered three times a day for seven days and Amoxicillin (500mg, Hakim Pharmaceutical Co.; Tehran; Iran) three times a day for 10 days [ 6 ]. The sutures were removed after 14 days. In the follow-up sessions (2,4,6 months after surgery day), the supragingival plaque was removed if present and oral hygiene training was re-administered as needed. 

### Clinical parameters

The clinical parameters were measured in all groups using the Williams probe (HU-Friedy; Chicago; IL; USA). These parameters included vertical clinical attachment level (VCAL) , the vertical probing depth (VPD) (the distance from the free gingival margin to the pocket depth) [ 28 ], gingival index (GI) (based on Silness and Loe index) [ 29 ], recession depth (REC) (distance from the CEJ to the free gingival margin measured in midbuccal) [ 7 ], horizontal probing depth (HPD) (horizontal penetration of periodontal probe in furcation area) [ 8 ]. The VPD, VCAL, GI, HPD, and REC were recorded at baseline (T0), the time of surgery (T1), 3 months (T2), and 6 months after surgery (T3). 

### PRGF preparation

The preparation of PRGF was performed immediately before surgery, according to Anitua [ 30 ]. Thus, the blood sample (20 ml) was taken from all patients before surgery, poured into 5-ml test tubes containing an anticoagulant (3.8% sodium citrate), and then centrifuged at 460 rpm for 8 min (PRGFEndoret System IV Biotechnology Institute; Vitoria, Spain). The resulting product contained layer 1 consisting of plasma (1ml) with small amounts of growth factor, layer 2 (PGF layer) with a volume of about twice as much as layer 1 with growth factors, layer 3 (PRGF layer) consisting of plasma (0.5ml) containing different growth factors; layer 4 (buffy coat layer) containing white blood cells (0.5 ml); and layer 5 containing red blood cells. A 500-µl pipette was used to take both layers 1 and 2; a 100-µl pipette was used to take the PRGF layer in five small aliquots to avoid mixing with the layer 4. Then, 1 ml of PRGF was added by 50µl of calcium chloride (10%), thereby activating the plasma rich in growth factor.

### ATV1.2% bio adhesive preparation

The biofilm or mucoadhesive patches were prepared by adding 95 cc of distilled water to 500cc of Erlenmeyer located on a warm plate stirrer. Next, the essential Carbopol [ 31 ] was added to water and heated. Afterwards, the methyl paraben and propyl paraben were solved in 95% of alcohol and then all solutions were added to the beaker as well. Next, the required amount of Glycerin was added to this solution. Finally, to prepare the bio adhesive, 0.5g of ATV per 100ml of gel was added [ 32 ]. 

### Radiological assessment

The first radiograph was provided by using a photostimulable phosphors (PSP) digital sensor size #2 (PCT; Soredex, Helsinki, Finland) with parallel technique. Bite registration was performed by using acrylic resin (Duralay, Reliance, Dental, Mgf company, Chicago, USA), to ensure having the same occlusion through the next radiographies. For the next radiographies, taken six months later, the same kV (kilo voltage), mA (milli Ampere), exposure time, and the same occlusion record were employed. Images were recorded as Digital Imaging and Communications in Medicine (DICOM) series and processed by Windows version 2.5 (PCT, Soredex; Helsinki, Finland). The digital subtraction of before and after treatment images was performed by using Adobe Photoshop (CC/2015.5.0 Release, San Jose, California, USA). 

### Statistical analysis

Quantitative data were recorded as Mean±Standard deviation. The mean comparisons were performed by the parametric tests (repeated measures ANOVA and one way ANOVA) and in the case of qualitative data non-parametric tests (Chi square test, Friedman, and Fisher's Exact Test). Data were analyzed by SPSS.23 (SPSS-Chicago; USA) at a significance level of *p*< 0.05.

## Results

In the present clinical trial, 15 patients were included and 24 sites intervened through open flap debridement / ATV/ PRGF/ ATV with PRGF, including 11 females and 4 males with a mean age of 42 years ranged from 35 to 46 years. No side effects such as wound opening and infection were observed during and after the operation.

### Radiological findings

Interpretations of radiographs are shown in Table 1. The changes in radiopacity were found in two cases of ATV with PRGF group, and the Chi-square test showed no difference among the study groups (*p*= 0.08).

**Table 1:** Radiographic changes of bone within each treatment group and comparison with other groups

Variable	Groups (n=24)			
	Control (n=6)	ATV (n=6)	PRGF (n=6)	ATV with PRGF (n=6)
Radiolucency	0	0	0	0
No change	6	6	6	4
Radio opacity	0	0	0	2
X2(2)= 6.545, *p* Value*=0.08				

*: Chi square test, ATV (Atorvastatin 1.2%), PRGF (plasma rich in growth factors)

### Clinical findings

Data of patients' clinical parameters are given in Tables 2, 3 and 4. There was no significant difference in the mean VPD, VCAL and HPD among the studied groups at T0, T1, T2 and T3 (Tables 2 [Table T3] to 4). At T2, the GI score was significantly different (*p*= 0.04) among the groups (Table 5). According to Tukey's test, the mean GI score in the ATV group was significantly lower than in the control group (*p*= 0.03) and the comparison between other groups was not significant.

**Table 2:** Vertical probing depth (VPD) changes within each treatment group and comparison with other groups

Groups	T0	T1	T2	T3	*p* Value***
Control (n=6)	5.33±0.81	4.33±0.51	4±0.63	3.5±0.54	0.001
ATV (n=6)	5±0.89	4.5±0.54	3.8±0.75	3.5±0.54	0.000
PRGF (n=6)	5.16±0.75	4.5±0.54	4±0.63	3.83±0.75	0.001
ATV with PRGF (n=6)	4.66±0.81	4.16±0.40	3.66±0.81	3.83±0.75	0.000
*p* Value	0.552*	0.599**	0.770**	0.497**	

*: ANOVA, **: Kruskal-Wallis Test, ***: Repeated measurement ANOVA; ATV(Atorvastatin 1.2%),PRGF(plasma rich in growth factors)

**Table 3 T3:** Vertical clinical attachment loss (VCAL) changes within each treatment group and comparison with other groups

Groups	T0	T1	T2	T3	*p* Value***
Control (n=6)	4.66±0.51	4.5±0.54	4.1±0.4	4±0.63	0. 292
ATV (n=6)	4.1±0.4	4.16±0.4	3.66±0.51	3.33±0.51	0.009
PRGF (n=6)	4.33±0.51	4.33±0.51	3.5±0.54	3.33±0.51	0.001
ATV with PRGF (n=6)	4±0.0	4±0.0	3.5±0.54	3.5±0.54	0.076
P value*	0.080	0.235	0.133	0.196	

*: Kruskal-Wallis Test, **: Repeated measurement ANOVA; ATV(Atorvastatin 1.2%),PRGF(plasma rich in growth factors)

**Table 4:** Horizontal probing depth (HPD) changes within each treatment group and comparison with other groups

Groups	T0	T1	T2	T3	*p* Value**
	Mean±SD	Mean±SD	Mean±SD	Mean±SD	
Control (n=6)	3.33±0.4	3.33±0.4	3.25±0.41	3.25±0.41	0.363
ATV (n=6)	3.33±0.51	3.33±0.51	3.08±0.37	2.83±0.4	-
PRGF (n=6)	3.5±0.54	3.5±0.54	3.5±0.54	3.5±0.54	0.062
ATV with PRGF (n=6)	3.25±0.41	3.25±0.41	3.08±0.66	3.08±0.58	0.694
*p* Value*	0.598	0.598	0.444	0.353	

*: Kruskal-Wallis Test, **: Repeated measurement ANOVA; ATV(Atorvastatin 1.2%),PRGF(plasma rich in growth factors)

**Table 5:** Gingival Index (GI) changes within each treatment group and comparison with other groups

Groups	T0	T1	T2	T3	*p* Value**
	Mean±SD	Mean±SD	Mean±SD	Mean±SD	
Control (n=6)	1.98±0.31	0.53±0.24	0.93±0.31	0.80±0.36	<0.001
ATV (n=6)	1.88±0.54	0.40±0.28	0.63±0.20	0.56±0.21	0.001
PRGF (n=6)	1.90±0.82	0.40±0.34	0.50±0.26	0.56±0.29	<0.001
ATV with PRGF (n=6)	1.90±0.47	0.55±0.26	0.65±0.18	0.50±0.20	<0.001
*p* Value*	0.990	0.283	0.043	0.693	

*: ANOVA, **: Repeated measurement ANOVA; ATV(Atorvastatin 1.2%),PRGF(plasma rich in growth factors)

Based on the repeated measurement ANOVA test, a statistically significant difference (*p*< 0.001) was observed in the VPD level among all groups from the baseline to the end line of the study (Table 2). In addition, regardless of the evaluation time, the mean VPD level was significantly different among the four groups (*p*< 0.001). However, the interaction between the VPD level and the groups had no significant difference (*p*> 0.05).

The repeated measurement ANOVA test showed a statistically significant difference (*p*< 0.05) in the VCAL level between the ATV and PRGF groups from the baseline to the end line of the study (Table 3). Furthermore, regardless of the evaluation time, the mean VPD level was significantly different among the four groups (*p*< 0.001). However, the interaction between the VCAL level and the group revealed no significant difference (*p*> 0.05).

According to the repeated measurement ANOVA test; there was no statistically significant difference (*p*> 0.05) in the HPD level among all groups from the baseline to the end line of the study (Table 4).

Regardless of the evaluation time, the mean HPD level was not significantly different among the four groups and the interaction between the HPD level and the group had no significant difference (*p*> 0.05).

The repeated measurement ANOVA test revealed a statistically significant difference (*p*< 0.001) in the GI score among all groups from the baseline to the end line of the study (Table 5). In addition, regardless of the evaluation time, the mean GI score showed a significant difference among the four groups (*p*< 0.001), but the interaction between the HPD level and the group had no significant difference (*p*> 0.05).

The REC parameter was compared among four study groups from T0 to T3 using Fisher's exact and Friedman tests (*p*> 0.05) (Table 6). 

**Table 6:** Recession depth (REC) changes within each treatment group and comparison with other groups

	REC	Control (n=6)	PRGF (n=6)	ATV (n=6)	ATV with PRGF (n=6)	Total	*p* Value*
T0	without recession	2 (33.3%)	2 (33.3%)	3 (50.0%)	3 (50.0%)	10 (41.7%)	1.000
	with recession	4 (66.7%)	4 (66.7%)	3 (50.0%)	3 (50.0%)	14 (58.3%)	
T1	without recession	4 (66.7%)	3 (50.0%)	2 (33.3%)	3 (50.0%)	12 (50.0%)	0.941
	with recession	2 (33.3%)	3 (50.0%)	4 (66.7%)	3 (50.0%)	12 (50.0%)	
T2	without recession	3 (50.0%)	5 (83.3%)	4 (66.7%)	5 (83.3%)	17 (70.8%)	0.766
	with recession	3 (50.0%)	1 (16.7%)	2 (33.3%)	1 (16.7%)	7 (29.2%)	
T3	without recession	3 (50.0%)	5 (83.3%)	5 (83.3%)	6 (100.0%)	19 (79.2%)	0.314
	with recession	3 (50.0%)	1 (16.7%)	1 (16.7%)	0 (0.0%)	5 (20.8%)	
*p* Value**		0.753	0.277	0.392	0.190		

*: Fisher's Exact Test, **: Friedman Test; Atorvastatin (ATV) 1.2%, Plasma Rich in Growth Factors (PRGF)

## Discussion

In the present study, we conducted the clinical and radiographic evaluation of PRGF along with ATV1.2% in the treatment of grade II furcation defects in the mandibular molars. We applied the combination of PRGF with ATV1.2% in furcation defects considering the higher benefits of PRGF compared to PRP systems. These included the ease of use, feasibility of usage at clinical office and hospital environments, simple tools, lower costs, minimized patients’ discomfort during bloodletting (owing to the need for a very small amount of blood), no possibility of infection, and minimum preparation time [ 7 ].

According to the results, there was no significant difference among the groups of PRGF, ATV, and PRGF with ATV with the flap group in terms of improvement of clinical parameters (REC, VCAL, VPD, and HPD). Nevertheless, improvement in each group was significant since the beginning to the end of the study. This lack of significance might be due to the complexity of the treatment of regeneration in the furcal region. Radiologically, increased radiopacity was observed in only two out of six cases in the ATV with PRGF group, which was not statistically significant. To the best of our knowledge, no clinical trial similar to our study has been conducted to date. However, many studies [ 6 - 8 , 22 , 28 ] have been performed on the use of PRGF along with different types of augmentation materials in the treatment of periodontal lesions, which have had similar results.

For instance, Mansouri *et al*. [ 6 ] evaluated the effect of bovine porous bone mineral along with PRGF on the treatment of grade II furcation defects, concluding that PRGF decreased GI, PD and CAL more than other groups. Nevertheless, their results were not statistically significant. In another study, Lafzi *et al*. [ 8 ] assessed the treatment of molars with grade II furcation involvement in two groups treading autogenous bone grafts with and without PRGF. These researchers reported significant improvement in V-CAL and a significant decrease in clinical PD and surgically exposed the horizontal probing depth of bony defect (E-HPD) at the end of the study. Nonetheless, the difference between the groups was not statistically significant. Similarly, we found better repair and improvement of clinical parameters in the group but no significant difference among the groups. In the current research, the examined parameters have been improved in the PRGF group at the end of the experiment, which was not statistically significant compared to the other groups.

On the other hand, Pradeep *et al*. [ 33 ] assessed the effect of PRP on the treatment of grade II furcation defects of mandibular molars, reporting that while the PRP group significantly improved at the end of the research, compared to the control group, the compound’s inability to completely cure furcation defects showed its limited role in the treatment of defects as a regenerative factor. Therefore, it is still early to make a definitive statement about the clinical and biological effects of the clinical use of PRGF. In another study, Pradeep *et al*. [ 22 ] applied Rosuvastatin (1.2 mg) in a combination of PRF and porous-hydroxyapatite (bone graft) in the treatment of grade II furcation defects. According to the results, clinical and radiographical parameters improved in the lesions treated, compared to open flap debridement.

Martande *et al*. [ 34 ] assessed the effect of ATV 1.2% combined with PRF on the treatment of intraosseous lesions in patients with chronic periodontitis. According to the results of this study, while the use of ATV and PRF had similar clinical improvement effects to the PRF-treated group alone, more improvements were observed in the radiographical parameters in the ATV and PRF group. In the current research, ATV 1.2% had positive effects on the improvement of clinical parameters. However, the results were insignificant and all our treatment groups had similar positive effects on clinical parameters.

In a research, Jenabian *et al*. [ 7 ] assessed the effect of PRGF and guided tissue regeneration (GTR) on the treatment of grade II furcation defects, reporting a significant improvement in clinical parameters of GI, VPD, and VCAL at the end of the research. 

In addition, Bojarpour *et al*. [ 28 ] evaluated the effect of PRGF and SRP on the treatment of periodontal three-walled intrabony defects, concluding that PRGF improved PPD but not GI. This lack of similarity between the studies might be due to different samples. In the current research, no significant difference was found among the groups in terms of all parameters assessed at T0 and T1. Therefore, the differences observed might be due to the type of treatment. One of the most important clinical changes in regenerative studies is the changes in PPD and CAL after regenerative treatment. In the present study, a VPD decrease was observed in all groups at the end of the experiment. In addition, there was a significant decrease in VCAL in the ATV and PRGF groups, compared to the beginning of the research. However, the difference between the groups was not statistically significant. This finding showed that the increase in attachment plays a major role in reducing the probing depth in both groups and the change in gingival margin position is a small part of the decrease. Attachment increase can be due to true periodontal regeneration or defect healing by new connective tissue attachment or long junctional epithelium [ 8 ]. Histological studies are needed to determine the nature of attachment enhancement. Furthermore, the decrease in VPD in the ATV group may be due to the fact that statins inhibit inflammatory cells and MMP levels, which related to PPD and bleeding during probing and play an important role in the regeneration of connective tissue in periodontal disease [ 25 ]. 

The positive role of statins in periodontal regenerative therapy has been shown in systematic reviews [ 35 - 36 ]. Similar to the present research, Shirke *et al*. [ 25 ] and Pradeep *et al*. [ 37 ] evaluated the effect of ATV 1.2% on the treatment of chronic periodontitis, finding that ATV could decrease PPD and CAL when concurrently used with SRP. 

In the present study, we evaluated the effect of the simultaneous use of ATV and PRGF Combination of these two substances showed synergistic effects in partial resolution of lesions on radiological exams in two out of the six samples, which was not statistically significant. This difference might be due to the small sample size recruited in the current research. We treated early grade II furcation defects and assumed that the remaining surrounding living tissues were able to provide a sufficient amount of periodontal target cells to be affected by growth factors present in PRGF and ATV. Future studies on larger sample sizes along with open flap debridement are recommended.

## Conclusion

Considering the limitations of the present study, PRGF along with ATV 1.2% was effective in the treatment of furcation defects in the mandibular molars and decreased GI, VPD and VCAL. However, there was no significant difference between this treatment modality and the other examined therapeutic items. 

## Conflict of Interest

The authors declare that they have no conflict of interest.

## References

[1] Mehta DB, Deshpande NC, Dandekar SA ( 2018). Comparative evaluation of platelet-rich fibrin membrane and collagen membrane along with demineralized freeze-dried bone allograft in Grade II furcation defects: A randomized controlled study. J Indian Soc Periodontol.

[2] Najim U, Slotte C, Norderyd O ( 2016). Prevalence of furcation‐involved molars in a Swedish adult population. A radiographic epidemiological study. Clin Exp Dent Res.

[3] Newman MG, Takei H, Klokkevold PR, Carranza FA (2019). Newman and Carranza's Clinical Periodontology E-Book.

[4] Troiano G, Laino L, Dioguardi M, Giannatempo G, Lo Muzio L, Lo Russo L ( 2016). Mandibular Class II Furcation Defect Treatment: Effects of the Addition of Platelet Concentrates to Open Flap: A Systematic Review and Meta-Analysis of Randomized Clinical Trials. J Periodontol.

[5] Inamdar MNK, Khan S, Ali SA, Ahmad E ( 2015). Management of Class-II Furcation Complicated with Endodontic involvement using Two Different Regenerative Materials. J Int Oral Health.

[6] Mansouri SS, Ghasemi M, Darmian SS, Pourseyediyan T ( 2012). Treatment of mandibular molar class II furcation defects in humans with bovine porous bone mineral in combination with plasma rich in growth factors. J Dent (Tehran).

[7] Jenabian N, Haghanifar S, Ehsani H, Zahedi E, Haghpanah M ( 2017). Guided tissue regeneration and platelet rich growth factor for the treatment of Grade II furcation defects: a randomized double-blinded clinical trial- a pilot study. Dent Res J (Isfahan).

[8] Lafzi A, Shirmohammadi A, Faramarzi M, Jabali S, Shayan A ( 2013). Clinical Comparison of Autogenous Bone Graft with and without Plasma Rich in Growth Factors in the Treatment of Grade II Furcation Involvement of Mandibular Molars. J Dent Res Dent Clin Dent Prospects.

[9] Camelo M, Nevins M ( 2000). Treatment of Class II Furcations with Autogenous Bone Grafts and e-PTFE Membranes. Int J Periodontics Restorative Dent.

[10] Upadhyay P, Blaggana V, Tripathi P, Jindal M ( 2019). Treatment of furcation involvement using autogenous tooth graft with 1‐year follow‐up: a case series. Clin Adv Periodontics.

[11] Khanna D, Malhotra S, Naidu DV ( 2012). Treatment of grade II furcation involvement using resorbable guided tissue regeneration membrane: A six-month study. J Indian Soc Periodontol.

[12] Christgau M, Moder D, Wagner J, Glassl M, Hiller KA, Wenzel A, et al ( 2006). Influence of autologous platelet concentrate on healing in intra-bony defects following guided tissue regeneration therapy: a randomized prospective clinical split-mouth study. J Clin Periodontol.

[13] Asimuddin S, Koduganti RR, Panthula VNR, Jammula SP, Dasari R, Gireddy H ( 2017). Effect of autologous platelet rich fibrin in human mandibular molar grade II furcation defects- a randomized clinical trial. J Clin Diagn Res.

[14] Tarallo F, Mancini L, Pitzurra L, Bizzarro S, Tepedino M, Marchetti E ( 2020). Use of platelet-rich fibrin in the treatment of grade 2 furcation defects: systematic review and meta-analysis. J Clin Med.

[15] Bajaj P, Pradeep AR, Agarwal E, Rao NS, Naik SB, Priyanka N, et al ( 2013). Comparative evaluation of autologous platelet-rich fibrin and platelet-rich plasma in the treatment of mandibular degree II furcation defects: a randomized controlled clinical trial. J Periodontal Res.

[16] Anitua E, Sanchez M, Orive G ( 2010). Potential of endogenous regenerative technology for in situ regenerative medicine. Adv Drug Deliv Rev.

[17] Verma PK, Srivastava R, Gupta K, Chaturvedi T ( 2013). Treatment strategy for guided tissue regeneration in various class II furcation defect: Case series. Dent Res J (Isfahan).

[18] Biswas S, Sambashivaiah S, Kulal R, Bilichodmath S, Kurtzman GM ( 2016). Comparative Evaluation of Bioactive Glass (Putty) and Platelet Rich Fibrin in Treating Furcation Defects. J Oral Implantol.

[19] Panda S, Karanxha L, Goker F, Satpathy A, Taschieri S, Francetti L, et al ( 2019). Autologous Platelet Concentrates in Treatment of Furcation Defects-A Systematic Review and Meta-Analysis. Int J Mol Sci.

[20] Mehta D, Deshpande N, Dave D, Modi B, Bharwani A ( 2016). Platelet rich fibrin: New treatment modality in Grade II furcation defects. J Oral Dis Marker.

[21] Garg S, Pradeep AR (2017). 1.2% Rosuvastatin and 1.2% atorvastatin gel local drug delivery and redelivery in the treatment of class II furcation defects: a randomized controlled clinical trial. J Periodontol.

[22] Pradeep AR, Karvekar S, Nagpal K, Patnaik K, Raju A, Singh P ( 2016). Rosuvastatin 1.2 mg In Situ Gel Combined With 1:1 Mixture of Autologous Platelet-Rich Fibrin and Porous Hydroxyapatite Bone Graft in Surgical Treatment of Mandibular Class II Furcation Defects: A Randomized Clinical Control Trial. J Periodontol.

[23] Anitua E ( 1999). Plasma rich in growth factors: preliminary results of use in the preparation of future sites for implants. Int J Oral Maxillofac Implants.

[24] Panda S, Purkayastha A, Mohanty R, Nayak R, Satpathy A, Das AC, et al ( 2020). Plasma rich in growth factors (PRGF) in non-surgical periodontal therapy: a randomized clinical trial. Braz Oral Res.

[25] Shirke PY, Kolte AP, Kolte RA, Bawanakar PV ( 2019). Evaluation of the clinical efficacy of 1.2% atorvastatin in the treatment of periodontal intraosseous defects by CBCT: A randomized controlled clinical trial. J Dent Res Dent Clin Dent Prospects.

[26] Kanoriya D, Bajaj N, Pradeep A ( 2019). 1.2% rosuvastatin gel as a local drug delivery agent in smokers with chronic periodontitis: a randomized controlled clinical trial. JCRI.

[27] Cao R, Li Q, Chen Y, Yao M, Wu Q, Zhou H ( 2019). Efficacy of locally-delivered statins adjunct to non-surgical periodontal therapy for chronic periodontitis: a Bayesian network analysis. BMC Oral Health.

[28] Bojarpour M, Jenabian N, Haghanifar S, Khafri S ( 2018). The effect of plasma rich in growth factors (PRGF) in the treatment of periodontal three-walled intrabony defects. Caspian J Dent Res.

[29] Löe H ( 1967). The Gingival Index, the Plaque Index and the Retention Index Systems. J Periodontol.

[30] Anitua E ( 2001). The use of plasma-rich growth factors (PRGF) in oral surgery. Pract Proced Aesthet Dent.

[31] Kumar K, Dhawan N, Sharma H, Vaidya S, Vaidya B ( 2014). Bioadhesive polymers: novel tool for drug delivery. Artif Cells Nanomed Biotechnol.

[32] Hashemi M, Ramezani V, Seyedabadi M, Ranjbar AM, Jafari H, Honarvar M, et al ( 2017). Formulation and Optimization of Oral Mucoadhesive Patches of Myrtus Communis by Box Behnken Design. Adv Pharm Bull.

[33] Pradeep AR, Pai S, Garg G, Devi P, Shetty SK ( 2009). A randomized clinical trial of autologous platelet-rich plasma in the treatment of mandibular degree II furcation defects. J Clin Periodontol.

[34] Martande SS, Kumari M, Pradeep AR, Singh SP, Suke DK, Guruprasad CN ( 2016). Efficacy of statin delivery as an adjunct to scaling and root planing in the treatment of chronic periodontitis: A meta-analysis. J Periodontol.

[35] Akram Z, Vohra F, Javed F ( 2018). Efficacy of statin delivery as an adjunct to scaling and root planing in the treatment of chronic periodontitis: A meta-analysis. J Investig Clin Dent.

[36] Sinjab K, Zimmo N, Lin GH, Chung MP, Shaikh L, Wang HL ( 2017). The Effect of Locally Delivered Statins on Treating Periodontal Intrabony Defects: A Systematic Review and Meta-Analysis. J Periodontol.

[37] Pradeep AR, Kumari M, Rao NS, Martande SS, Naik SB ( 2013). Clinical efficacy of subgingivally delivered 1.2% atorvastatin in chronic periodontitis: a randomized controlled clinical trial. J Periodontol.

